# The Potential Clinical Benefit of Tocilizumab Therapy for Patients with HHV-8-infected AIDS-related Multicentric Castleman Disease: A Case Report and Literature Review

**DOI:** 10.7759/cureus.7589

**Published:** 2020-04-08

**Authors:** Gauri Barlingay, Dawood Findakly, Carlos Hartmann, Surabhi Amar

**Affiliations:** 1 Internal Medicine, Creighton University Arizona Health Education Alliance/Valleywise Health, Phoenix, USA; 2 Internal Medicine, Creighton University Arizona Health Education Alliance/Valleywise Health Medical Center, Phoenix, USA; 3 Infectious Disease, Ochner Health System, Covington, USA; 4 Hematology/Oncology, Creighton University Arizona Health Education Alliance/Valleywise Health, Phoenix, USA; 5 Oncology, University of Arizona College of Medicine - Phoenix Campus, Phoenix, USA

**Keywords:** tocilizumab, anti-interleukin 6, multicentric castleman disease, angiofollicular lymph node hyperplasia, acquired immunodeficiency syndrome, kaposi sarcoma, hhv-8, case report, literature review

## Abstract

Castleman disease (CD), also known as angiofollicular hyperplasia, is a rare disorder characterized by nonmalignant mediastinal lymph node enlargement provoked by excess interleukin-6 (IL-6) secretion. It could be unicentric or multicentric (MCD). Here, we describe a 27-year-old man with a prior history of AIDS, Kaposi sarcoma (KS), and latent syphilis who presented to the ED for persistent fatigue, fever, chills, night sweats, and productive cough. Infectious workup was negative, and the patient continued to have a high fever despite empiric antibiotic therapy. Bone marrow biopsy was performed and was negative for malignancy. The patient eventually underwent a left clavicular lymph node biopsy, which showed a plasma cell variant CD with positive immunostaining for human herpesvirus 8 (HHV-8), and high HHV-8 viral load. We started the patient on rituximab and liposomal doxorubicin, but unfortunately, the patient had a severe anaphylactic reaction to the rituximab, so we could not proceed with this treatment. We, therefore, started tocilizumab treatment, which improved the patient's general condition, and he was eventually discharged from our hospital. Upon follow-up 11-months later, a repeat CT scan of the chest and abdomen showed a near-complete treatment response with decreased lymphadenopathy throughout and hepatosplenomegaly. IL-6 overproduction in patients with CD is linked to the production of inflammatory cytokines and has a role in tumor angiogenesis, which makes it potential for IL-6 targeted therapy. The diagnosis of CD, especially MCD, requires a high index of suspicion, and a lymph node biopsy is essential in the diagnosis. Tocilizumab, an IL-6 receptor antibody, could potentially be considered as a practical therapeutic approach in managing HHV-8 positive MCD patients who do not tolerate or respond to initial rituximab therapy.

## Introduction

Castleman disease (CD) is a rare systemic condition characterized by nonmalignant mediastinal lymph node enlargement prompted by excess interleukin-6 (IL-6) secretion. Patients infected with human herpesvirus 8 (HHV-8), also known as Kaposi sarcoma (KS)-associated herpesvirus infection, could be associated with multicentric CD (MCD) [1]. We typically see MCD in older adults except in HIV-positive patients, where it presents at a younger age [2]. MCD characteristically presents with a spectrum of constitutional symptoms including fever, night sweats, unintentional weight loss, fatigue, and body aches. Systemic signs of both progressive generalized lymphadenopathies and hepatosplenomegaly [3]. HIV-positive and HHV-8 positive patients often develop KS and are more prone to developing HIV-related lymphoproliferative disorders. MCD has variable clinical outcomes, and HHV-8 co-infecting HIV-positive MCD patients are found to be associated with poor outcomes [4-6]. Rituximab is the most commonly used therapy for MCD. Unfortunately, to date, there is no standard therapy for patients who fail or cannot tolerate rituximab-based therapies. Patients with MCD may benefit from treatment with an anti-IL-6/IL-6 receptor therapy. Studies are continuing to investigate tocilizumab as an acceptable alternative therapy to rituximab for HIV-1-positive and HHV-8-positive MCD [7]. Here, we report a young HIV positive patient who presented with recurrent high fevers and was diagnosed with MCD. Our patient could not tolerate rituximab therapy, and therefore, tocilizumab, an IL- 6, was administered with a near-complete response to treatment.

## Case presentation

**Figure 1:**
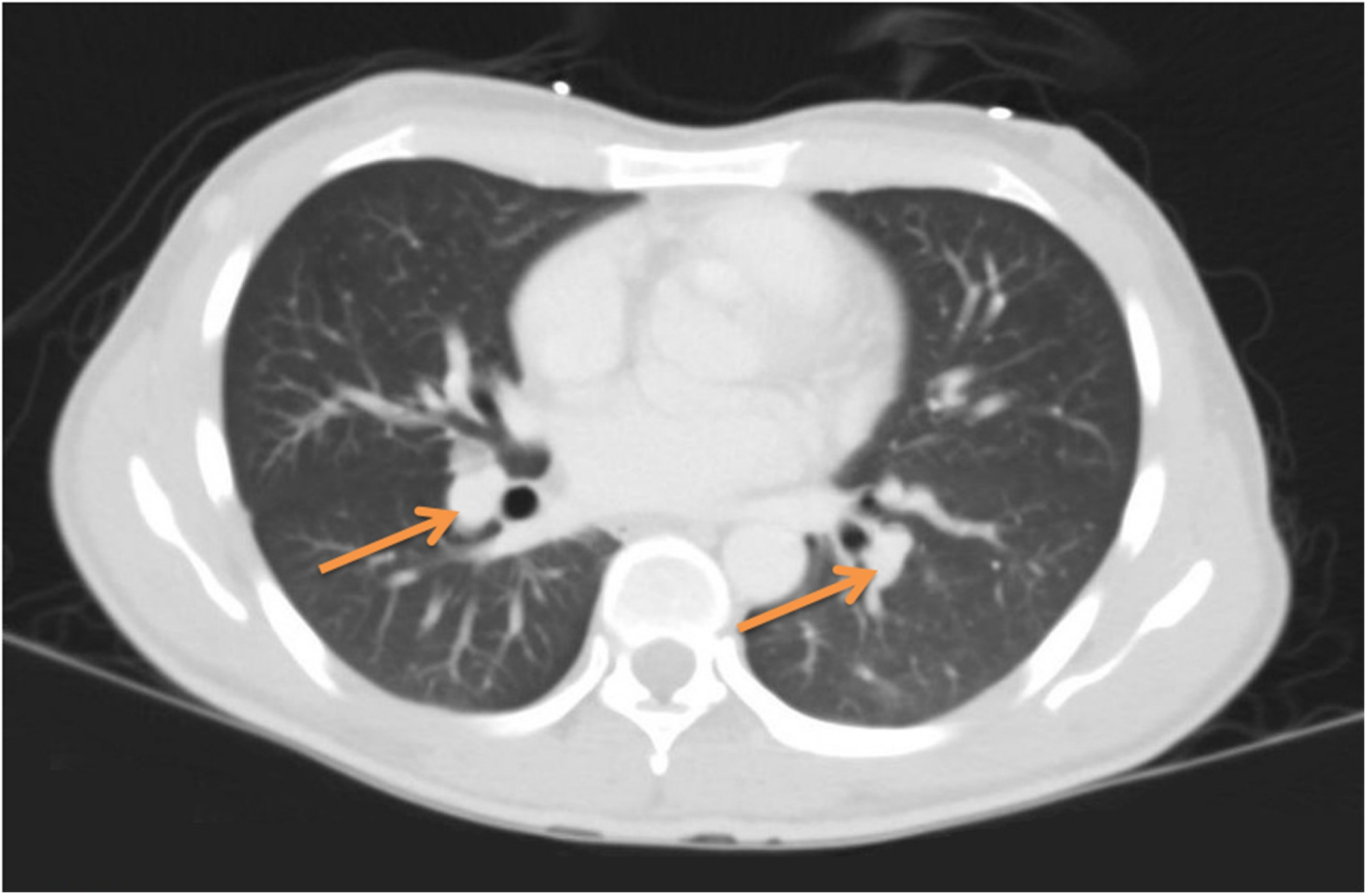
Chest CT scan showing prominent aortopulmonary lymph nodes (arrows).

A 27-year-old man with a past medical history significant for AIDS, KS, and latent syphilis came to the ED complaining of persistent fatigue, fever, chills, night sweats, diffuse headaches, and a productive cough with white sputum. He was found to have a persistent temperature of up to 40.1 degrees Celsius, and tachycardia. His initial physical exam demonstrated fine crackles at the bilateral lower lung lobes. His initial laboratory workup revealed leukocytosis with a white cell count of 5.6 x 109/L, mild anemia with a hemoglobin of 10.6 g/dL, thrombocytopenia with a platelet count of 61 × 109/L, coagulopathy with a prothrombin time of 19.6 s, INR of 1.8, normal lactic acid at 0.9 mmol/L, and elevated pro-calcitonin at 1.95 μg/L. A chest X-ray revealed prominent pulmonary interstitial markings. CT chest, abdomen, and pelvis showed hepatosplenomegaly with diffuse lymphadenopathy, which was concerning for malignancy and, or disseminated infection (Figure 1). 

**Figure 2:**
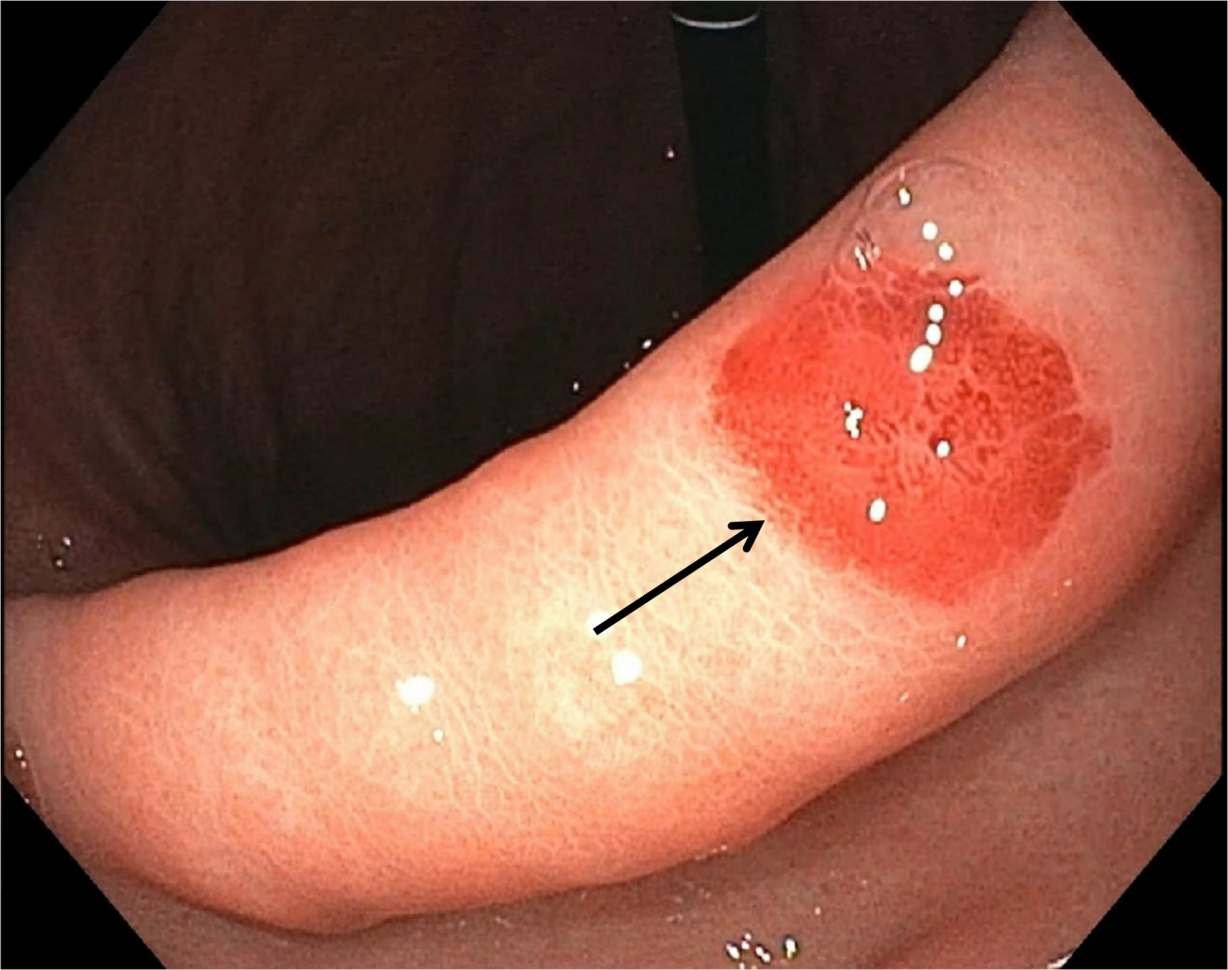
EGD showing an 0.8 cm hyperemic patch along the lesser curvature of the stomach (arrow).

**Figure 3:**
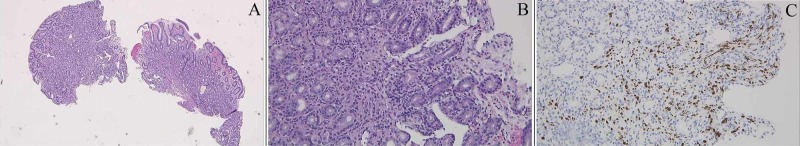
Gastric KS. (A) KS composed of spindle cell proliferation involving lamina propria (hematoxylin and eosin staining; magnification, ×40). (B) gastric mucosa with a focal area of spindle cell proliferation with extravasated red blood cells involving the lamina propria and cells have enlarged hyperchromatic nuclei (magnification, ×200). (C) An immunohistochemical stain for HHV-8 shows positive staining. KS, Kaposi sarcoma; HHV-8, human herpesvirus 8

Initially, his presentation was thought to be due to sepsis of unknown etiology. Therefore, empiric therapy with piperacillin-tazobactam, linezolid, acyclovir, and fluconazole was started. Infectious diseases and hematology-oncology teams were consulted to aid in the patient's management. Despite empiric therapy, the patient continued to have high daily fevers and night sweats. Subsequently, antibiotics were changed to vancomycin and ceftriaxone. Infectious workup, including coccidiosis-specific antibodies and cerebrospinal fluid testing, returned negative, and therefore, fluconazole and acyclovir were discontinued. Meanwhile, during admission, the patient developed diarrhea and pancytopenia for which cytomegalovirus (CMV) colitis was diagnosed and treatment with valganciclovir initiated. Esophagogastroduodenoscopy (EGD) was performed, showing an 0.8 cm hyperemic patch along the lesser curvature of the stomach (Figure 2). A biopsy from the lesion was taken, and a single new gastric KS was seen after the histopathological examination (Figure 3A,B). The immunoperoxidase stain was positive for HHV-8 (Figure 3C).

**Figure 4:**
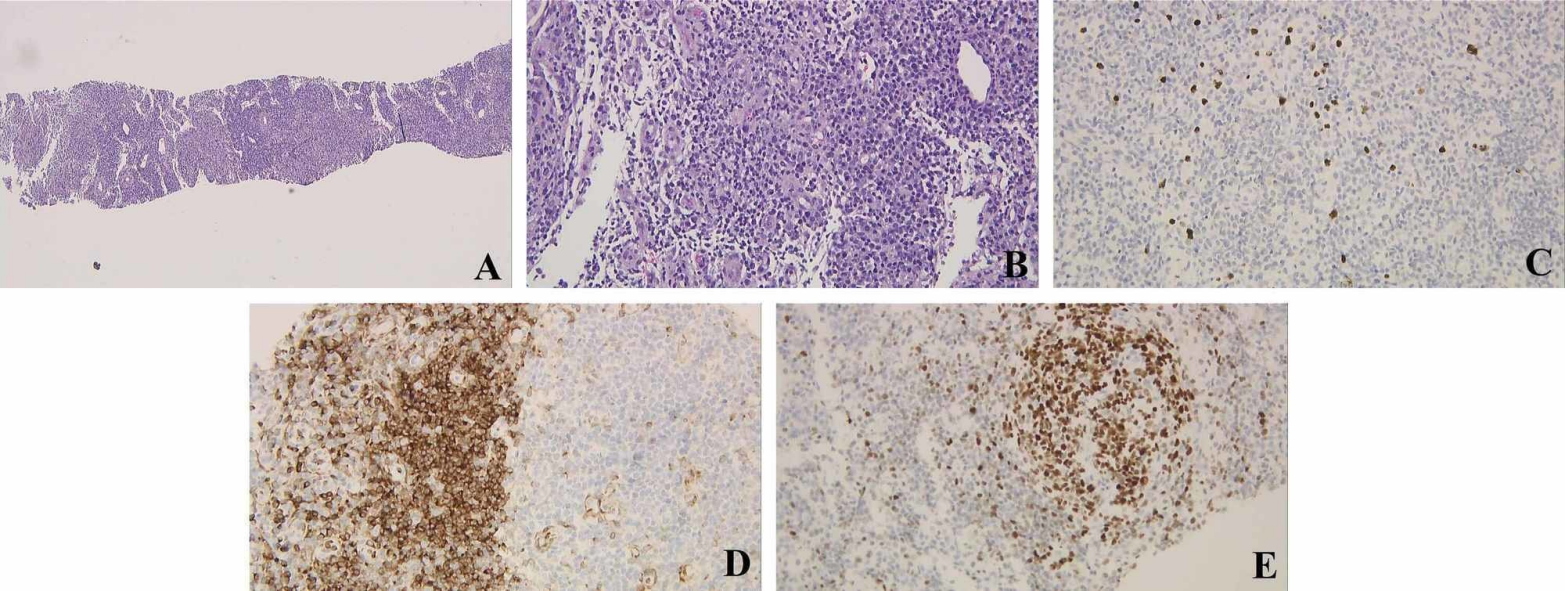
Left clavicular LN needle core biopsy. (A) LN tissue (hematoxylin and eosin staining; magnification, ×40). (B) Mildly increased vascularity in the interfollicular areas, sheets of mature plasma cells, and lymphoid follicles with germinal centers are present (magnification, ×200). (C) Immunoperoxidase stain for HHV-8 shows positive staining of interfollicular cells (magnification, ×200). (D) Bcl-2 stain negative (magnification, ×200). (E) BCL-6 stains the germinal centers positively (magnification, ×200). LN, lymph node; HHV-8, human herpesvirus 8; BCL, B-cell lymphoma

Throughout his stay, labs were pertinent for anemia, which required blood transfusions, hypergammaglobulinemia, elevated C-reactive protein (CRP), low cluster of differentiation 4 (CD4) count of 173 cells/mm3, undetectable HIV RNA, and an elevated procalcitonin. Bone marrow biopsy was performed and was negative for malignancy, and no organisms identified on lymph node biopsy. Moreover, blood and urine cultures described no growth. The patient eventually underwent a left clavicular lymph node biopsy, which showed a plasma cell variant CD with B-cell lymphoma (Bcl)-2-negative, Bcl-6-positive, and positive immunostaining for HHV-8 with a high HHV-8 viral load at 196 copies/mL (Figure 4A-E).

He was started on rituximab and liposomal doxorubicin for MCD and KS, respectively. Unfortunately, the patient had a severe anaphylactic reaction to rituximab, and this recurred despite pretreatment with dexamethasone; hence we could not proceed with this treatment. Then, tocilizumab therapy was considered, and upon initiation of therapy, the patient's symptoms, vital signs, and laboratory derangements began to improve. Subsequently, the patient was discharged from the hospital on tocilizumab therapy and was prescribed valganciclovir for his CMV. Repeat CT scans showed a near-complete treatment response with decreased lymphadenopathy throughout and hepatosplenomegaly. His laboratory values continued to improve upon follow-up. Since his discharge home 14 months ago, the patient has not been re-admitted to the hospital, and he continues to follow-up regularly with his HIV specialist and the oncology clinic. He has tolerated the tocilizumab well, and his symptoms have entirely resolved.

## Discussion

Castleman disease is a rare nonmalignant lymphoproliferative disorder that is characterized by different clinical and histological subtypes. Clinically, it is divided into two types; either unicentric or MCD. Different histological subtypes have been described based on histological findings; those include the hyaline vascular (HV) type, the plasma cell (PC) type, and the mixed cell (MC) type. The HV type usually is asymptomatic, whereas the other types and the MCD type are usually associated with constitutional symptoms including fever, night sweats, weight loss, and fatigue. Labs are usually pertinent for anemia, thrombocytopenia, hyper-gammaglobulinemia, elevated hepatic transaminases, and IL-6 [8-9]. IL-6 implicated to cause constitutional symptoms and anemia in MCD through the production of inflammatory cytokines and hepatic hepcidin, respectively. Studies demonstrated IL-6 role in tumor angiogenesis, which is an essential potential in IL-6 targeted therapy [1]. Serum IL-6 level usually correlates with the degree of lymph node hyperplasia, hypergammaglobulinemia, acute-phase protein levels, and clinical characteristics. B-cell differentiation, acute-phase protein regulation, and immunoglobulin production are triggered by the hyperplastic lymph nodes' germinal centers. Isolated IL-6 overproduction by the germinal centers of the hyperplastic lymph nodes is found in patients with CD. Surgical removal of the individual hyperplastic lymph node was found to result in decreased cytokine production and clinical improvement [10].

The increasing incidence of HIV patients with MCD does not correlate with the patients' CD4 count or with the use of HAART [11]. MCD can be HHV-8 related (usually associated with generalized lymphadenopathy and hepatosplenomegaly) or HHV-8 unrelated [12]. Many patients have an initially low CD4 count, and it can often be confused for opportunistic infections. As the symptoms are vague, patients are often misdiagnosed or have recurrent admissions for systemic inflammatory response syndrome (SIRS). The diagnosis of CD, especially MCD, requires a high index of suspicion [13]. A lymph node biopsy will usually demonstrate a plasmacytic or plasmablastic response, which is consistent with our patient's presentation [14].

The usual treatment for HIV-1 positive and HHV-8 positive MCD is rituximab, a monoclonal anti-CD20 antibody. The KS can flare with the treatment of MCD; hence usually, KS treatment has to be restarted [15]. This guideline-directed therapy initially started in our patient. However, he had an anaphylactic reaction to rituximab despite premedication with decadron, so it was discontinued. Our patient is positive for both HIV-1 and HHV-8. Siltuximab, a monoclonal antibody to IL-6, was considered as a possible treatment option, but it has not been studied in HHV-8 positive patients. Furthermore, tocilizumab, on the other hand, is an IL-6 receptor antibody found to alleviate symptoms in HHV-8 positive MCD. In a clinical trial by Nishimoto et al., 28 patients with CD were treated with tocilizumab at a dose of 8 mg/kg IV every two weeks with reported improved inflammatory markers at six weeks of treatment. At four months of therapy, patients were found to have improved symptoms, reported less fatigue, had weight gain, and increased hemoglobin level. Furthermore, 36% of patients had improved hepatosplenomegaly and decreased lymph node size [16]. This dose was used in the treatment of our patients.

The lytic viral replication of HHV-8 is more common in MCD as opposed to in KS. It is thought that IL-6 is expressed mostly during this lytic phase, and so anti-viral therapy could potentially be used in patients with detectable HHV-8 viral loads. However, anti-viral treatment does not yet have a defined role in the treatment of MCD. One clinical trial showed the benefit of using high dose zidovudine and valganciclovir with a complete symptomatic response in more than 50% of patients who were HHV-8 positive [17]. Patients with HHV-8 positive MCD are more prone to developing non-Hodgkin's lymphoma and large B-cell lymphomas. In a single-center prospective cohort study published, it was found that HIV positive patients with MCD who were treated with rituximab therapy had an 11-fold lower risk of developing lymphoma [18]. No data exist on whether tocilizumab therapy would also protect against future HIV-associated lymphomas in MCD patients.

Our patient was treated with tocilizumab and was continued on valganciclovir as an anti-herpes virus agent, which has been used to treat symptomatic HIV positive and HHV-8 positive patients. We noticed an excellent response to this therapy, which was not only seen in the remarkable improvement in his functional status but also noticeable by the near-complete treatment response upon repeat CT scans of the chest, abdomen, and pelvis with decreased both lymphadenopathy and hepatosplenomegaly. His lab markers (CRP) also improved significantly, and he is back to his baseline functional status.

## Conclusions

The MCD patients who cannot tolerate or are refractory to first-line therapy constitute a real challenge to clinicians. Our patient successfully sustained remission with tocilizumab therapy after not tolerating rituximab therapy. This case suggests a potential therapeutic role of the anti-IL6 receptor antibody, tocilizumab, as a treatment modality in AIDS-associated HHV-8-infected MCD patients.
